# A case report of intracranial hemorrhage after spinal anesthesia

**DOI:** 10.1186/s40981-017-0081-x

**Published:** 2017-03-09

**Authors:** Yuri Iwase, Manzo Suzuki, Hiroyasu Bito

**Affiliations:** Department of Anesthesiology, Musashikosugi Hospital Nippon Medical School, 1-396 Kosugi-cho Nakahara-ku, Kawaski, Kanagawa 211-8533 Japan

**Keywords:** Chronic subdural hematoma, Postdural puncture headache, Combined spinal and epidural anesthesia, Aspirin

## Abstract

**Background:**

Chronic subdural hematoma (CSDH) after spinal anesthesia is a rare complication. We experienced a patient who developed CSDH after postdural puncture headache (PDPH) following combined spinal and epidural anesthesia (CSE).

**Case presentation:**

A 38-week-gestation parturient with a history of previous cesarean delivery underwent elective cesarean section under CSE. She had been receiving aspirin therapy for Kawasaki disease for many years. She developed a symptom of PDPH 1 day after the surgery. Fluid administration and analgesics were started. Although the headache was relatively severe and persistent, it suddenly disappeared on the third postoperative day. Aspirin administration was restarted on the third postoperative day, and the patient was discharged 1 week after the surgery. 2 weeks after being discharged, she was readmitted to our hospital for severe headache and was diagnosed as having CSDH. An epidural blood patch was performed, resulting in resolution of the hematoma.

**Conclusions:**

We experienced a case of CSDH after PDPH in a patient who was receiving aspirin therapy. Aspirin therapy should be restarted after confirmation of the absence of headache. We should consider the possibility of unexpected disappearance of PDPH in the postoperative period may be due to the development of CSDH.

## Background

Postdural puncture headache (PDPH) is a benign condition and a frequent complication associated with neuraxial anesthesia. Intracranial chronic subdural hematoma (CSDH) is a rare but lethal complication following PDPH. More than 50 cases of subdural hematoma after neuraxial anesthesia or dural puncture have been reported [1, 2]. We experienced a case of CSDH after cesarean delivery.

## Case presentation

A 34-year-old parturient with a history of previous cesarean delivery underwent elective cesarean delivery at 38 weeks gestation. She was taking aspirin, 100 mg per day, for Kawasaki disease. The results of preoperative blood examination including platelet count, prothrombin time, and activated prothromboplastin time, were normal. After one-week cessation of aspirin therapy, an uneventful cesarean delivery was performed under combined spinal and epidural anesthesia (CSEA). Epidural catheterization was performed using an 18-gauge Tuohy needle (UNIVER, UNISYS, Saitama, Japan) through the Th12/L1 interspace. A catheter for epidural anesthesia was inserted without any complications. Spinal anesthesia was performed using a 25-gauge Quincke needle (UNISYS) at the L/3/4 interspace. A bolus of isobaric 0.5% bupivacaine, 2.2 ml, was injected, and a dermatomal level of sensory block at the level of Th10 was confirmed by alcohol swab. During the surgery, 0.25% levobupivacaine, 5 ml, was administered through the epidural catheter. After the surgery, a dermatomal level of sensory block at the level of Th6 was confirmed. Postoperative analgesia was obtained by epidural infusion of 0.25% levobupivacaine containing 10 μg of fentanyl at the rate of 4 ml/h. 1 day after the surgery, she had an occipital headache while in the sitting position with neck stiffness and back pain. Intravenous fluid administration, 1000 ml/day, and administration of loxoprofen, 60 mg three times a day, were started the day after surgery. Although the headache was relatively severe and seemed to be persistent, her headache suddenly disappeared on the third postoperative day. The patient recovered and she could walk around the ward on the fifth postoperative day. Administration of aspirin was restarted on the third day after the surgery by her obstetrician, and the decision to restart aspirin therapy was not based on disappearance of PDPH but just on their routine procedure. 1 week after the surgery, the patient was discharged from the hospital without significant symptoms.

**Fig. 1:**
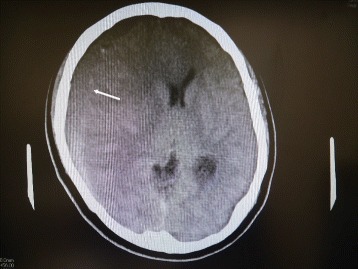
Computed tomogram obtained at the time of readmission to the emergency room. A subdural hematoma (*arrow*) is seen

**Fig. 2:**
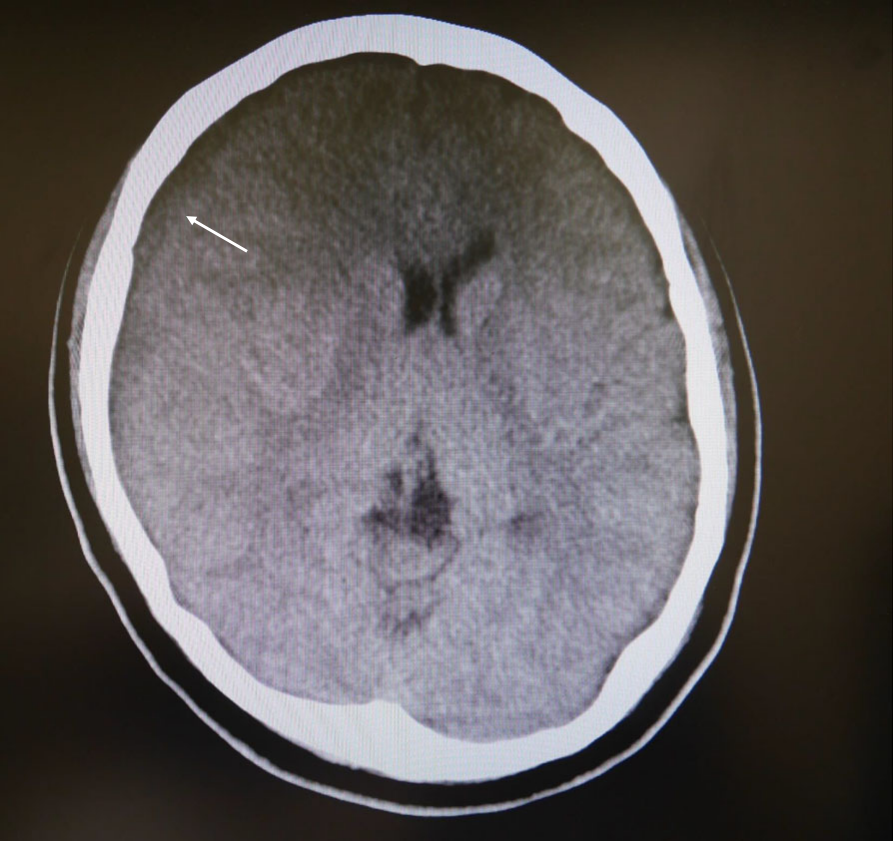
1 week after epidural blood patch was performed, computed tomogram shows resolution of the subdural hematoma (*arrow*)

2 weeks after hospital discharge, she presented to the emergency room with a severe headache, the severity of which was not related to her position. She complained that light headache had reappeared 3 days after being discharged, and the headache worsened over the next 10 days. Computed tomography (CT) showed an ipsilateral chronic subdural hematoma (Fig. 1). The patient was admitted to the hospital and aspirin therapy was stopped. While she had been staying in bed in the hospital, she complained of having a light headache without neurological deficits. After one-week cessation of aspirin administration, an epidural blood patch was performed. Surgical resection of the hematoma was scheduled as a back-up plan in case her headache became severe or neurological symptoms developed. During injection of 15 ml of blood in the L4/5 interspace using an 18-gauge Tuohy needle, she complained of light headache, which disappeared 1 h after the procedure. She was told to stay in bed for 1 week after the epidural blood patch. One week after the procedure, CT showed resolution of the hematoma (Fig. 2). Administration of aspirin was restarted 9 days after the procedure. The patient was discharged from the hospital 13 days after the procedure. The patient has not developed any further symptoms.

## Discussion

We experienced a patient who developed CSDH after combined spinal and epidural anesthesia for cesarean delivery. She developed a headache 1 day after the surgery which was suspected as being PDPH since she also had neck stiffness and back pain. However, the possibility of acute subdural hematoma soon after the surgery remained. Macon et al. [3] stated that a headache that persists and is more severe than PDPH may be due to acute subdural hematoma. Yildirim et al. [4] reported a patient with acute subdural hematoma that had been misdiagnosed as eclampsia. The development of an acute subdural hematoma soon after delivery presents a very fast time course and results in coma [4–6]. In the present case, although her headache was relatively severe and seemed to be persistent, it disappeared suddenly on the third postoperative day. This time course including temporal improvement of the headache suggested that the headache was not due to development of acute intracranial hemorrhage soon after the surgery, but that it was due to dural puncture.

Many case reports suggested the mechanism of the development of CSDH following PDPH [1, 2, 7]. Some reports suggested that temporal disappearance of headache or a fatal outcome was due to development of a hematoma [2, 4]. Leakage of cerebrospinal fluid (CSF) from a dural hole leads to reduced volume of CSF and results in a decrease in intraspinal and intracranial pressure. The change in cerebrospinal dynamics induces caudal movement of the spinal cord and brain which results in stretching of the dura, cranial nerve, and bridging vein, which in turn causes headache and bleeding from the vein [7]. The increase in intracranial pressure by the progression of intracranial hematoma may compensate the decrease in intracranial pressure by spinal fluid leakage. The unexpected disappearance of PDPH in the present case may have been due to progression of the formation of an intracranial lesion. Nakannuno demonstrated that 40% of case in CSDH after PDPH presented the time course which includes temporal disappearance of headache such as present case [1].

In several cases, administration of antiplatelets or anticoagulants had been suspected to be related to the development of PDPH [8–10]. Landman et al. [9] reported a case of CSDH in a patient who had self-administered aspirin and caffeine for the treatment of PDPH. There are two case reports of CSDH after improvement of PDPH in patients who received heparin infusion for thromboprophylaxis [10, 11]. In a parturient who developed CSDH after PDPH following cesarean delivery, the physicians hesitated in administering an epidural blood patch because she had thrombocytopenia due to HELLP syndrome [12]. In the present case, the patient had been taking aspirin for Kawasaki disease, and aspirin administration was restarted on the third postoperative day. Meta-analyses that examined both the primary and the secondary benefits of aspirin indicated that the risk of intracranial bleeding increased in a trial examining the primary benefit of aspirin in preventing cardiovascular disease, whereas the risk of intracranial bleeding did not increase in a trial that demonstrated the secondary benefit of aspirin in preventing cardiovascular disease [13, 14]. In healthy women, administration of aspirin does not increase the risk of intracranial bleeding [15]. One article that estimated the pooled relative risk of intracranial bleeding, demonstrated that long-term administration of aspirin increased the risk of intracranial bleeding, although the risk was still small [16]. However, among patients who developed intracranial bleeding, patients who had been taking aspirin possessed high mortality [17]. In the present case, even though the patient’s headache had improved at the time of restarting aspirin therapy, there remained a possibility that the improvement was a result of compensation of intracranial hypotension by the formation of a hematoma, and administration of aspirin may have worsened the degree of bleeding. Even though administration of aspirin may not have been the direct cause of the development of bleeding because aspirin was not administered in the perioperative period, the development of an antiplatelet effect may have contributed to the development of the hematoma. In the presence of intracranial hypotension, patients who are taking aspirin may be at high risk for intracranial bleeding.

In the present case, epidural blood patch was administered for the treatment of CSDH. Various treatments for CSDH after PDPH have been reported including observation, surgical resection of the hematoma, epidural blood patch, or both surgical resection and epidural blood patch [7, 8, 18, 19]. The treatment plan is decided according to the patient’s neurological symptoms and the size of the hematoma. In the present case, the patient complained of headache without neurological deficits, thus epidural blood patch was performed, considering surgical resection as a back-up plan. In the case of CSDH after PDPH, there is a possibility of recurrence of bleeding by only surgical drainage because the underlying cause of intracranial hypotension remains [20].

The guidelines of the American Society of Regional Anesthesia state that aspirin administration does not have to be suspended prior to administration of neuraxial anesthesia, in cases where aspirin is self-administered [21]. However, this recommendation is based on the assumption that a spinal or epidural hematoma develops only around the site of spinal or epidural puncture. Although the development of CSDH after PDPH following neuraxial anesthesia is rare, it is sometimes fatal. In the present case, the timing of when to restart aspirin therapy had been decided by her obstetrician. In present case, an anesthesiologist may be better to be involved in the decision of restart in antiplatelet therapy. This case report indicates that antiplatelet and anticoagulation therapy should be restarted after confirming that the patient does not have PDPH following neuraxial anesthesia. Antiplatelet and anticoagulation therapy should also be restarted after confirming that unexpected disappearance of PDPH is not due to the development of CSDH by CT imaging.

## Conclusions

We experienced a case of CSDH after PDPH in a patient who underwent neuraxial anesthesia. The patient was receiving aspirin therapy for Kawasaki disease. She underwent surgery after cessation of aspirin therapy for 1 week. It may be better to restart aspirin therapy after confirming the absence of PDPH after surgery. We should consider the possibility of unexpected disappearance of PDPH in the postoperative period may be due to the development of CSDH. Although incidence of CSDH after PDPH is rare, we believe that we may be better to confirm the absence of CSDH by CT before restart of aspirin therapy.

## Abbreviations

CSDH: Chronic subdural hematoma
CSE: Combined spinal and epidural anesthesia
CSEA: Spinal and epidural anesthesia
CSF: Cerebrospinal fluid
CT: Computed tomography
PDPH: Postdural puncture headache
